# Deviatoric stress-induced metallization, layer reconstruction and collapse of van der Waals bonded zirconium disulfide

**DOI:** 10.1038/s42004-024-01223-1

**Published:** 2024-06-22

**Authors:** Linfei Yang, Junwei Li, Dongzhou Zhang, Yuegao Liu, Qingyang Hu

**Affiliations:** 1 Center for High Pressure Science and Technology Advanced Research, 100193 Beijing, China; 2 School of Materials Science and Engineering, Jingdezhen Ceramic University, Jingdezhen, 333403 Jiangxi China; 3 Hawai’i Institute of Geophysics and Planetology, School of Ocean and Earth Science and Technology, University of Hawai’i at Manoa, Honolulu, HI 96822 USA; 4 CAS Key Laboratory for Experimental Study under Deep-sea Extreme Conditions, Institute of Deep-sea Science and Engineering, Chinese Academy of Sciences, Sanya, 572000 China; 5 Shanghai Advanced Research in Physical Sciences (SHARPS), Shanghai, 201203 China

**Keywords:** Two-dimensional materials, Solid-state chemistry, Electronic materials

## Abstract

In contrast to two-dimensional (2D) monolayer materials, van der Waals layered transition metal dichalcogenides exhibit rich polymorphism, making them promising candidates for novel superconductor, topological insulators and electrochemical catalysts. Here, we highlight the role of hydrostatic pressure on the evolution of electronic and crystal structures of layered ZrS_2_. Under deviatoric stress, our electrical experiments demonstrate a semiconductor-to-metal transition above 30.2 GPa, while quasi-hydrostatic compression postponed the metallization to 38.9 GPa. Both X-ray diffraction and Raman results reveal structural phase transitions different from those under hydrostatic pressure. Under deviatoric stress, ZrS_2_ rearranges the original ZrS_6_ octahedra into ZrS_8_ cuboids at 5.5 GPa, in which the unique cuboids coordination of Zr atoms is thermodynamically metastable. The structure collapses to a partially disordered phase at 17.4 GPa. These complex phase transitions present the importance of deviatoric stress on the highly tunable electronic properties of ZrS_2_ with possible implications for optoelectronic devices.

## Abstract (web summary)

Transition metal dichalcogenides exhibit rich polymorphism, making them promising candidates for superconductors, topological insulators, and electrochemical catalysts. Here, the authors study the metallization of non-hydrostatically compressed ZrS_2_ up to 45.8 GPa and find that a new high-pressure phase forms under deviatoric stress.

## Introduction

The discovery of graphene has ignited a fervent pursuit of novel two-dimension (2D) materials with exotic optical and electronic properties. Among the myriad candidates, transition metal dichalcogenides (TMDs) have drawn substantial attention due to their remarkable polymorphism and highly tunable band-gap structures. Their electronic structures were reported to span the entire range of solid states, encompassing Mott insulating^1,2^, semiconducting^3^, semimetallic to metallic^4^, and even superconducting states^5,6^. Such versatility of electronic property stems from their unique atomic arrangements, characterized by the stacking of sandwiched X-M-X monolayer (X: chalcogen, M: transition metal) interacted through weak van der Waals (vdW) forces^7,8^. To our best knowledge, three fundamental crystal symmetries were summarized for TMDs and they govern the interlayer stacking: 1 *T* (trigonal), 2*H* (hexagonal), and 3 *R* (rhombohedral), where the numbers of 1, 2, 3 denote the number of layers per unit cell. The interplay between the strong ionic-bonded interlayered structure and the weak vdW interaction give rise to the complex polymorphism under varying thermodynamical conditions.

Phase transitions of TMDs are induced by various external stimuli, such as mechanical exfoliation^9^, transverse electric field^10^ and intercalation^11^. Among these methods, applying pressure is a powerful and clean approach to modulate the electronic and crystal structures of TMDs without introducing impurities^12–14^. Taking the prototypal 2*H* phase (e.g. MoS_2_, MoSe_2_, MoTe_2_, WS_2_ and WSe_2_) as an example^15–19^, phase transitions under pressure are widely reported to be triggered by layer sliding or interlayer compression. A case in point is 2*H*-MoS_2_, which undergoes an isostructural phase transition from 2*Hc* to 2 *Ha* via the lateral displacement of adjacent atom layers, accompanied by metallization driven by the reduction of interlayer spacing under compression^20^. Such an interlayer phase transition does not alter the arrangement of intralayer atoms, their coordination number, or the unit cell volume. Since the vdW force is typically associated with the softest vibrational modes and collapse prior to strong interlayer bonding, lattice reconstruction within the vdW layer is rarely observed in pressurized systems. However, polymorphism within low dimensions has emerged as the origin of novel superconducting and topological states^21,22^, potentially useful for making optics and optoelectronic devices.

Unlike the 2*H* polytype, the 1 *T* phase has even richer structure polymorphism and exhibits distinct physical behaviors under pressure. This work focuses on the semiconducting 1 *T* phase ZrS_2_, which was previously predicted to transform to a metallic phase by applied pressure of only 5.6 GPa^23^. However, optical transmittance measurements indicated that it keeps the insulator state at least up to 15.4 GPa^24^. This contrast of experimental results on the electronic structure encourages us to conduct direct electrical measurements to clarify the controversy. In addition, theoretical studies suggest that the six-fold ZrS_6_ octahedron at ambient conditions is inherently unstable and transforms to ZrS_10_ bicapped cuboids under pressure^23^. The abrupt coordination change in 1*T*-ZrS_2_ is an anomaly among conventional AB_2_-type TMDs and implies the existence of metastable structures with unique structural motifs.

In this work, we provide evidence for the onset of metallization in nonhydrostatic compressed ZrS_2_ at above 30.2 GPa. Using the same pressurization protocol, a first-order structural transition within the 1*T*-ZrS_2_ layer without breaking the weak vdW interaction was observed at 5.5 GPa. The layered structure was observed to maintain until 17.6 GPa, where an isostructural phase transition occurred and formulated a partially disordered structure. At the meantime, our parallel experiments conducted under quasi-hydrostatic conditions showed that both metallization and structural phase transition pressures were substantially postponed.

## Results and discussion

### Electronic phase transition

**Fig. 1:**
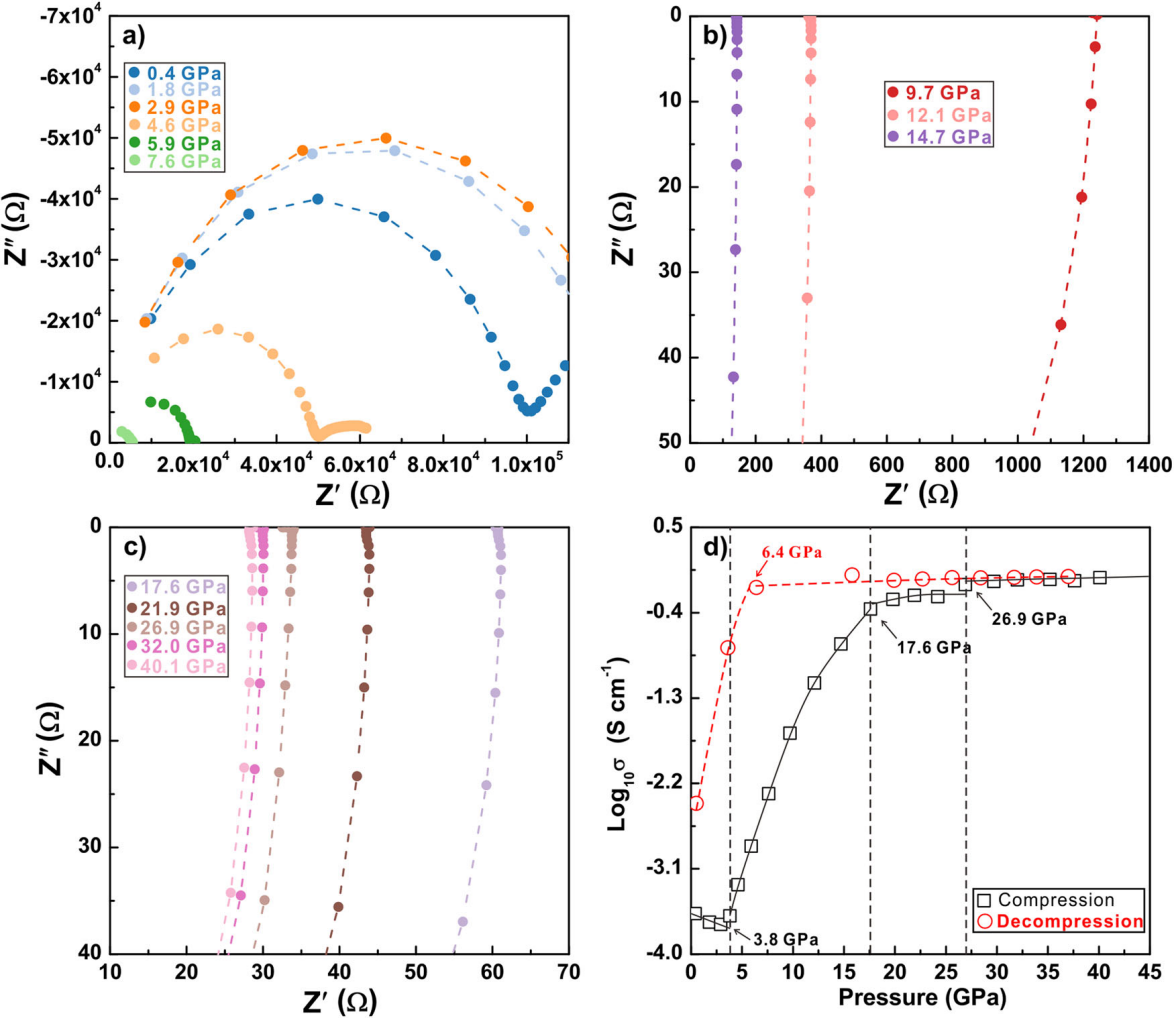
In situ electrical conductivity results at room temperature and high pressures. **a–c** Typical impedance spectra for ZrS_2_ under non-hydrostatic compression in the frequency region of 10^‒1^–10^7 ^Hz during pressurization. The horizontal axe indicates the real part of the complex impedance, while vertical axe represents the imaginary part. **d** The logarithm of electrical conductivity as a function of pressure during the process of compression and decompression. The error bars are within in data points.

The electronic properties of ZrS_2_ under various hydrostatic conditions and room temperature were investigated up to 40.1 GPa. In our electrical conductivity experiment, the sample was connected by two Pt probes to measure the in situ AC impedance under high pressure, which has been widely employed in the measurement of electrical conductivity of materials^25–27^. For non-hydrostatic pressure (Fig. 1), the evolution of impedance can be divided into three sections. For the ambient stable phase (namely phase I), the electrical conductivity of ZrS_2_ was about 2.68 (13)×10^-4 ^S/cm, which is the typical value of semiconductor. The EC value slightly decreased upon pressurization 3.8 GPa, above which the EC jumped due to promoted charge carrier mobility (named as phase II). The electrical conductivity reached a plateau at above 17.6 GPa (phase III). Comparing to the EC at ambient conditions, the phase III has achieved roughly four orders of magnitude enhancement. The EC remained a high value of (e.g. 0.9 S/cm at 26.9 GPa) to the highest pressure studied, thus corresponding to a metallic phase. At the plateau, the increase rate of carrier concentration with pressure would be substantially reduced with the progression of pressure-induced metallization. We then decompressed the sample to examine the reversibility of the aforementioned transition. The EC had remained high level until 6.4 GPa (Fig. 1d), where EC rapidly dropped and approach semiconductor values. The hysteresis of transition pressure during the compression/decompression cycle is a common phenomenon for first-order transition. After decompression to 1 atm, the EC value is 3.89 (21)×10^-3 ^S/cm, which is higher than the original state at ambient condition. This unrecoverable EC value indicates the structural phase transition is irreversible.

The same experiments were also conducted under quasi-hydrostatic conditions (Supplementary Fig. 1). Using KCl as solid pressure media, the phase transition boundary to the phase II and the onset of metallization are both shifted to higher pressure. Specifically, the first transition point (8.2 GPa) from phase I to phase II is much higher than that (3.8 GPa) under non-hydrostatic compression. We note that at 8 GPa, KCl was previously measured to has pressure gradient approximately of 0.6 GPa, 70% lower than the one without pressure media^28^. At the meantime, the EC values increase much more slowly, reaching 0.84 S/m at 38.9 GPa. In comparison, our non-hydrostatic experiment has similar level of EC at early as 30.0 GPa, where we regarded it as metal. The delay of the first-order transition and metallization is possibly due to the protection of pressure medium. These results by varying the hydrostatic conditions are consistent with previous studies of compressed TMDs, such as WS_2_ and MoSe_2_^19,27^.

**Fig. 2:**
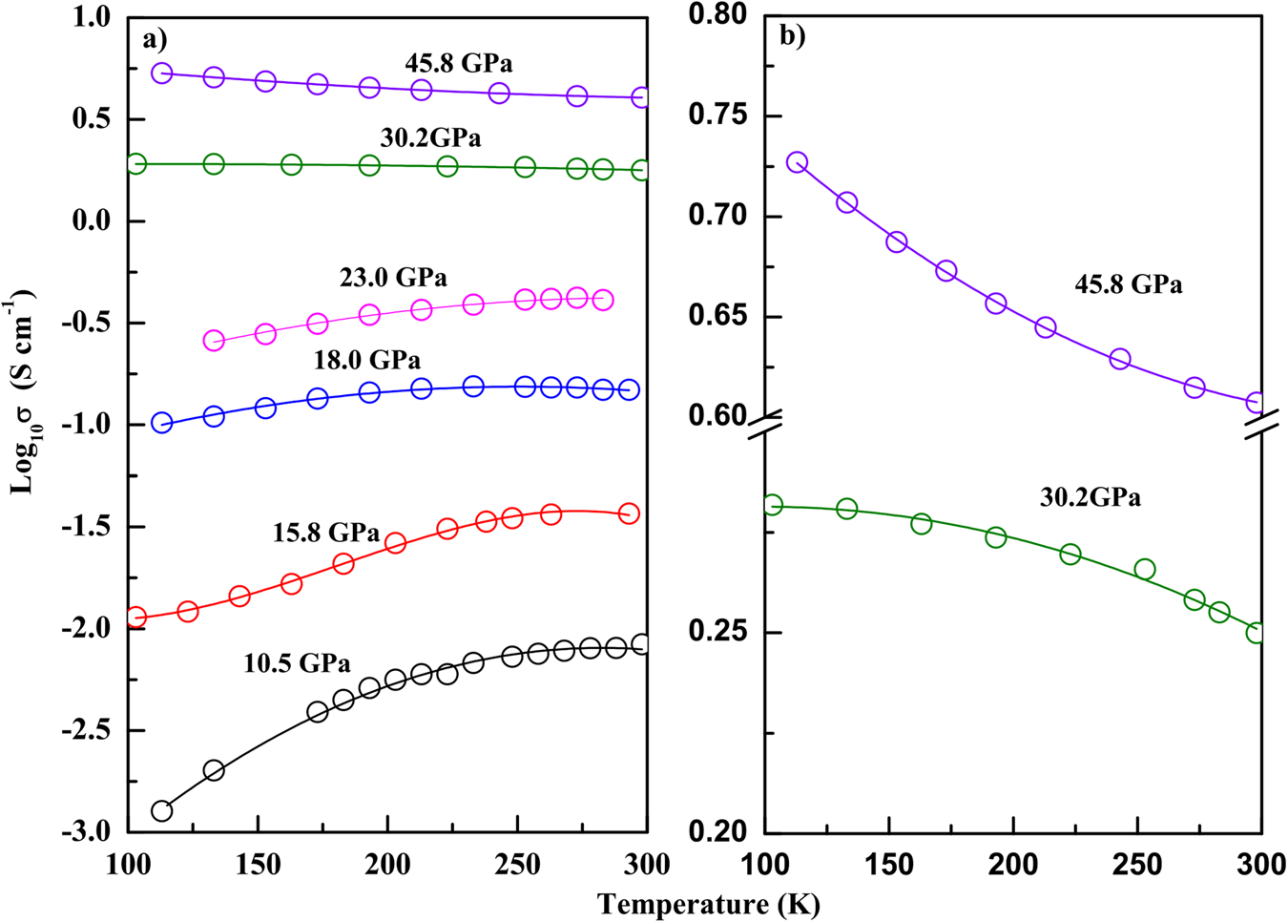
Electrical conductivity results at low temperatures and high pressures. **a** The temperature dependence of the electrical conductivity (EC) of ZrS_2_ at various pressure points. **b** The metallization at 30.2 GPa and 45.8 GPa.

We further measured the temperature dependence of EC (Fig. 2) under non-hydrostatic conditions to diagnose the semiconductor-to-metal transition. For typical insulator and semiconductors, the EC exhibits a positive correlation with temperature^29,30^. In our experiments, the slope of log10(*σ*)/*T* approach zero between 23.0 and 30.2 GPa, indicating the pressure range within which metallization occurs. Our observed temperature dependence confirms that the phase II is still a semiconductor and pressure-induced metallization occur in phase III. The four-order-of-magnitude increase in EC during compression makes ZrS_2_ a promising material for designing optoelectronic switches controlled by pressure.

### Crystal structural transition by XRD and Raman

**Fig. 3:**
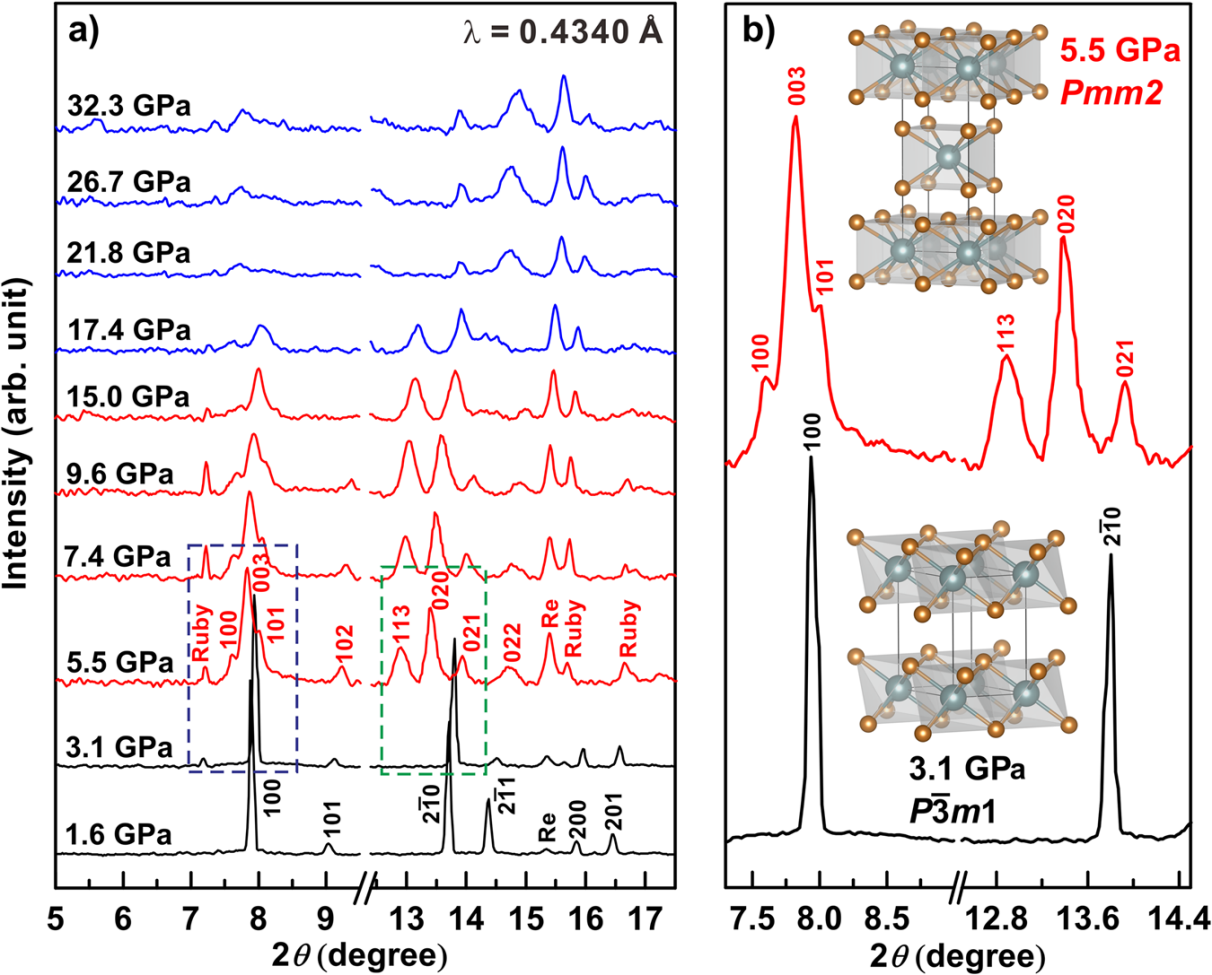
The evolution of XRD pattern of ZrS_2_ at elevated pressures and room temperature. The X-ray wavelength is 0.4340 Å. **a** XRD patterns up to 32.3 GPa with breaks between 9.5 to 12.4 degrees. The breaks are for clarity purpose because the omitted diffraction angles are dominated by the gasket signals. Peaks of Re gasket and ruby are labeled in the figure. Full spectra at 1.6 and 15.0 GPa were shown in Supplementary Fig. 6. **b** Comparison of the enlarged XRD data at 3.1 GPa and 5.5 GPa within the dashed blue and green boxes in (**a**), and the occurrence of first-order structural phase transition is clearly indicated at 5.5 GPa. The Miller indices (hkl) are drawn in diagram for $P{\bar{3}}m1$ and *Pmm*2 phases. The inset denotes the crystal structure of the $P{\bar{3}}m1$ and *Pmm*2 phases.

The electronic properties hinge to the crystal structure and therefore we performed in situ synchrotron XRD experiments of compressed ZrS_2_ up to 32.3 GPa. In order to obtain a comparable result with our EC measurements, no pressure medium was used in XRD experiments. Figure 3a displayed representative XRD patterns during the compression. The diffraction patterns at pressures below 5.5 GPa are readily indexed to the trigonal phase ($P\bar{3}m1$, phase I) as illustrated in the inset of Fig. 3b with Re gasket and minor amount of ruby. Although there are some differences in the XRD spectrum at 1.6 GPa (Fig. 3a) and atmospheric pressure (Supplementary Fig. 2), most diffraction peaks are consistent. All of Bragg peaks for the trigonal ZrS_2_ shifted to higher angles with increasing pressure, indicating conventional compression behavior. Pronounced changes in XRD pattern were observed when pressure was increased to 5.5 GPa, at which several new diffraction peaks appeared at 2*θ* angles of around 7.6°, 7.8°, 8.0°, 12.9°, 13.4° and 13.9°, suggesting the onset of structural phase transition toward to phase II. We observed the disappearance of several diffraction peaks when pressure increased above 17.4 GPa, leaving only four broad peaks (Supplementary Fig. 3). At this point, the crystalline feature of the sample began to diminish, and a degree of structural disordering (denoted by phase III) came into play. The limited number and severe broadening of diffraction peaks in phase III preclude the determination of its lattice parameters.

We employed a Monte Carlo indexing algorithm^31^ to identify the lattice associated with the newly appeared diffraction peaks of phase II and refined its space group using the Crysfire software package^32^. The indexed twelve XRD peaks for this high-pressure phase were listed in Table 1 and the diffraction pattern was further refined in Supplementary Fig. 4. The results pointed to an orthorhombic structure with the space group of *Pmm*2 with lattice parameters *a* = 3.297 (6) Å, *b* = 3.627 (7) Å, *c* = 9.509 (11) Å and *V* = 56.85 (34) Å^3^ at 5.5 GPa. Although the *Pmm*2 was not predicted as a stable phase by previous structural searching simulation^23^, it can be realized as a conjugate subgroup of the host tetragonal structure (*I*4/*mmm*), which was previously regarded as the ground state at above 25 GPa. Such lattice distortion was well documented for phase transitions under spontaneous strain^33^. Martino et al.^24^ also observed a structural phase transition in compressed ZrS_2_ occurring at about 3.0 GPa, but determined a different structure phase (*P2*_*1*_*/m*). Such difference was possibly due to the effect of the deviatoric stress, which would alter the transition pathway and this effect is well-known in 2D van der Waals layered materials^19,26,27^. In this study, the deviatoric stress would be inevitably generated in the sample chamber due to the lack of pressure medium. To quantify the deviatoric stress, we placed several small rubies in the center and at the edge of the sample chamber to reflect pressure gradient. As shown in Supplementary Fig. 5, the difference of pressure from the center to the edge of sample chamber gradually increase under compression. The results on the effects of hydrostatic conditions are reproducible to our impedance, XRD and the following Raman measurements.

**Table 1:** The indexed XRD peaks for Phase II at 5.5 GPa

Phase II (5.5 GPa)	*h*	*k*	*l*	2*θ* (degree)	2*θ* (degree)	*Δ* 2*θ*
				experiment	refined	
Orthorhombic (*Pmm*2) *a* = 3.297 (6) Å *b* = 3.627 (7) Å *c* = 9.509 (11) Å *V* = 56.85 (34) Å^3^	1	0	0	7.599	7.6048	–0.0058
	0	0	3	7.818	7.8478	–0.0298
	1	0	1	8.012	8.0428	–0.0308
	1	0	2	9.226	9.2337	–0.0077
	1	0	3	10.975	10.9363	0.0387
	1	1	3	12.885	12.9028	–0.0178
	0	2	1	13.919	13.9264	–0.0074
	0	1	5	14.733	14.7837	–0.0507
	2	0	1	15.443	15.4685	–0.0255
	0	2	4	17.275	17.2546	0.0204
	2	1	4	19.809	19.7664	0.0426
	1	0	7	19.935	19.9125	0.0225

**Fig. 4:**
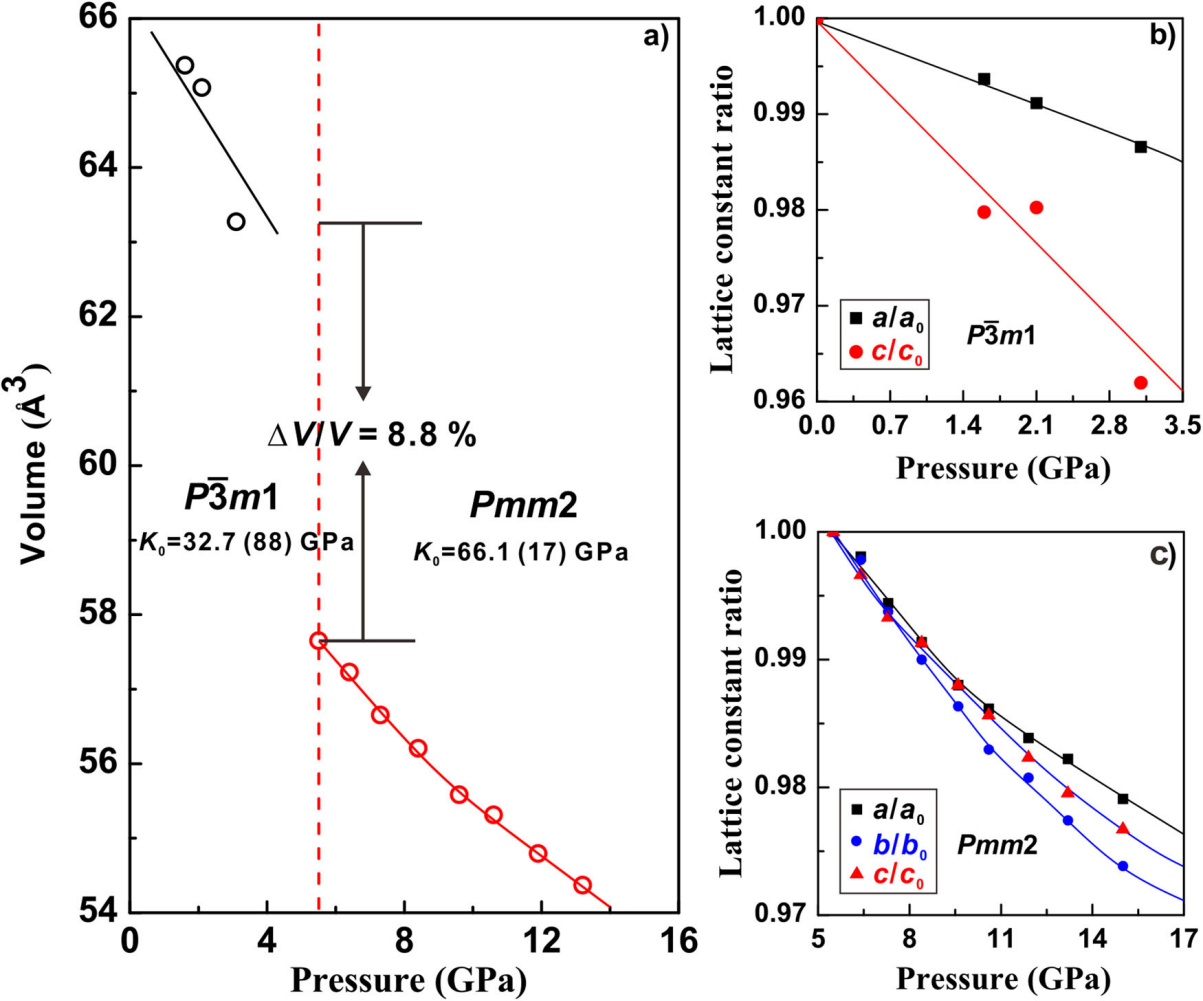
The unit cell volume and lattice parameter ratios with increasing pressure. **a** The pressure-dependent unit cell volume of ZrS_2_ for $P\bar{3}m1$ phase (black circle) and *Pmm*2 phase (red circle) in the pressure range from 0–16 GPa. The solid curves represent the fitting results with BM equation of state. The vertical dashed lines denote the transition points at 5.5 GPa (red). **b**, **c** The lattice parameter ratios of ZrS_2_ as a function of pressure for $P\bar{3}m1$ and *Pmm*2 phases.

The evolutions of unit cell volume with pressure of ZrS_2_ are plotted in Fig. 4a. Upon the transformation from $P\bar{3}m1$ to *Pmm*2 phase, the unit cell volume abruptly dropped by 8.8%, echoing its first-order transition nature from EC measurement. The pressure-volume data for different phases were fitted using a third-order Birch-Murnaghan equation of state in EosFit7 program^34^. The derived bulk modulus *K*_0_ and zero-pressure unit cell volume *V*_0_ for phase I are 32.7 (88) GPa and 68.6 (10) Å^3^, respectively. As for phase II, we acquired the bulk modulus *K*_0_ = 66.1 (17) GPa, and *V*_0_ = 61.3 (1) Å^3^, indicating that phase II is less compressible. The pressure dependence of lattice parameter ratios is illustrated in Fig. 4b, c. For the uniaxial compressibility of phase I, the *c* axis is more compressible than the *a* axis due to the weak vdW forces existed in the *c* axial orientation. In contrast, the high-pressure phase II exhibited much weak anisotropy in comparison with the $P\bar{3}m1$ phase.

The substantial volume collapse and severe lattice distortion associated with the I-II phase transition makes it impracticable for the precise determination of atomic coordinates. However, considering Phase II as a distorted tetragonal phase with two formula units in a conventional unit cell, and assuming the atomic coordinates from the undistorted tetragonal phase^23^, each Zr atom would be coordinated by 8 neighboring S atoms, forming ZrS_8_ cuboids due to the elongation of *b* axis. This distortion maintains a substantial interlayer distance (4.75 Å at 5.5 GPa), Suggesting that the phase II may retains a layered structure. In particular, the interlayer structural transition in ZrS_2_ is readily compared with other AB_2_-type TMDs with similar lattice structure, such as MoS_2_, WS_2_, and WSe_2_^16,35,36^, all of which are reported to undergo pressure-induced phase transition through layer sliding. Their structural transitions are associated with the lateral shift of adjacent atom layers, and are isostructural transition, which remain the layered nature^35^. However, under deviatoric stress, the phase transition of ZrS_2_ reconstructed the interlayer lattice to form an orthorhombic structure and the coordination number of Zr promoted from six to eight. The partially disordered phase III may eventually collapse phase II into a 3D structure. The postponed layer sliding in ZrS_2_ is possibly resultant from the relatively weak in-plane bonding in ZrS_2_.

**Fig. 5:**
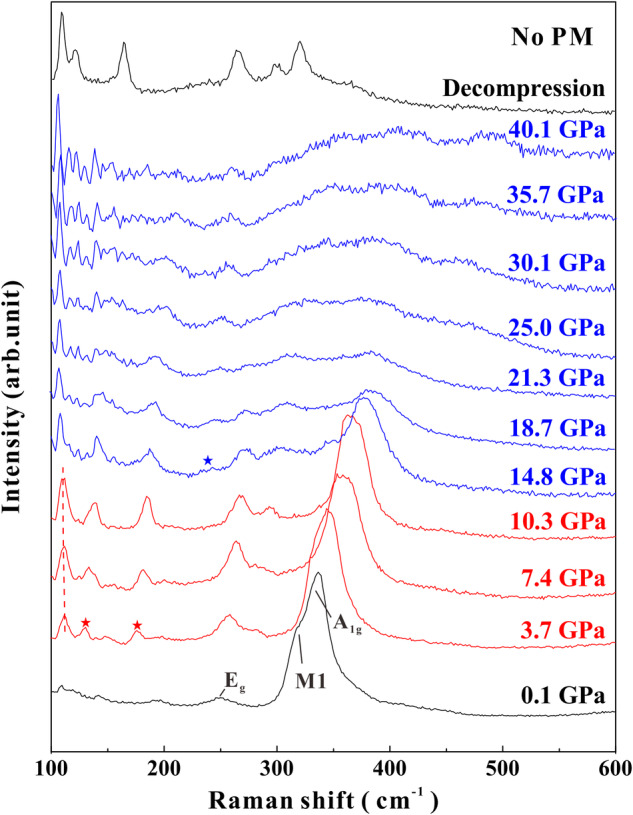
Raman spectra of ZrS_2_ as a function of pressure under non-hydrostatic conditions. No PM denotes without using pressure medium. The dashed line indicates the softening phonon mode in phase II. The pentagram marks the appearance of new peaks.

We further conducted Raman spectroscopy under non-hydrostatic and quasi-hydrostatic conditions with focus on the interlayer structure. Theoretical group analysis of lattice vibrations of 1*T*-ZrS_2_ at the *Γ*-point predicted two Raman-active modes represented as *Γ* = *A*_*1g*_ + *E*_*g*_ + 2*A*_*2u*_ + 2*E*_*u*_^37^. At ambient conditions, the in-plane (*E*_*g*_) and out-of-plane (*A*_*1g*_) Raman active modes of ZrS_2_ were clearly captured at positions of 248.8 cm^−1^ and 332.7 cm^−1^ (Fig. 5), respectively. A broadening peak near the A_1g_ band appeared at 312.7 cm^-1^ (M1), which can be explained by the non-harmonic effect induced by acoustic phonon despite coincidence with infrared-active A_2u_ mode, as was concerned in some literature^38^. All of these peaks obtained from this study are in good accordance with previous Raman data^38,39^.

Figure 5 present Raman spectra under deviatoric stress up to 40.1 GPa. Two inflection points were determined at 3.7 GPa and 14.8 GPa, which are verified by the appearance of new Raman peaks. These variations are corresponding to I-II and II-III phase transitions, reaching agreement with our synchrotron XRD data and EC results. In phase II, we noticed that Raman active modes at 111.5 cm^–1^ are featured by mild softening with negative pressure coefficient. This phonon softening is likely interrelated with structural phase transitions in ZrS_2_. Typical TMDs like ReS_2_^40^ were also found to exhibit phonon softening under high pressure. In addition, it can be clearly observed that the Raman signals in phase III are pretty weak above 30.1 GPa. In general, ideal metals do not have Raman-active phonon modes due to the screening from free charge, which would result in the obvious decrease in the Raman intensity^41^. Therefore, our non-hydrostatic data from Raman and EC experiments provide robust evidences for the deviatoric stress induced metallization in phase III of ZrS_2_.

In comparison with Raman spectra under non-hydrostatic conditions, the transition point from ZrS_2_ I to II occurred at higher pressure of 5.4 GPa under hydrostatic conditions (Supplementary Fig. 7) and the delay of transition is due to the protection of pressure medium, which have been widely reported in literature^19,25,26^. We compare Raman spectra for non-hydrostatic pressure of 10.3 GPa (no PM) and hydrostatic pressure of 10.9 GPa (with PM) in Supplementary Fig. 8. The Raman spectra of phase II under hydrostatic conditions showed a weak shoulder peak at 383.3 cm^–1^, which did not occur under non-hydrostatic conditions. Also shown in the inset figure Supplementary Fig. 8, the EC value of phase II under non-hydrostatic conditions is much higher than that under hydrostatic conditions, implying the evolution of crystal structure are deeply modulated by the applied deviatoric pressure.

## Conclusions

In summary, we have conducted a comprehensive suite of high-pressure experiments in DACs up to 45.8 GPa under non-hydrostatic compression, showing different results from those obtained under hydrostatic pressure. Using deviatoric stress, a new high-pressure phase III with partially disordered structure was unveiled above 17.4 GPa. This disordered phase III would become a metallic phase upon further compression. The identified metallic phase III with disordered structure in ZrS_2_ might provide helpful insight into the high-pressure behaviors of other similar layered TMDs compounds.

Our results also unambiguously revealed a first-order structural phase transition in ZrS_2_ at 5.5 GPa (phase II). In contrast to MoS_2_, this phase transition is caused by the intralayer reconstruction rather than the interlayer sliding, highlighting the fundamental differences in their bonding networks. The stability field of the ambient stable phase I is also expanded under deviatoric stress. For experiments conducted by using inert gas as pressure medium^24^, phase I transits to a monoclinic structure at 3.0 GPa. In short, applying deviatoric stress has engineered the transition pathway of phase transition. The pressure-quenchable, vdW-interacted phase II expands the polymorphic complexity of TMDs and may open avenues for further exploration in the design Zr-based dichalcogenides for optoelectronic implications.

## Methods

### Sample characterization

High-quality ZrS_2_ powder with purity of 99.99% was commercially acquired from Sunano company, Shanghai, China. The sample was ground into powders with grain size of ~1 μm by the agate mortar, and the same starting sample were used throughout our high-pressure experiments. The initial powdered sample with grain size of ~1 μm was characterized by an X’ Pert Pro X-ray powder diffractometer with the copper Kα radiation. The X-ray diffraction pattern (Supplementary Fig. 2) indicated the ZrS_2_ powder belongs to the trigonal lattice (space group: $P\bar{3}m1$) at ambient condition. Rietveld refinement was implemented in GSAS software^42^ to obtain lattice constants and unit cell volume as follows: *a* = *b* = 3.660 (1) Å, *c* = 5.833 (2) Å, and *V* = 67.676 (6) Å^3^.

### High-pressure electrical conductivity experiments

High-pressure electrical conductivity (EC) measurements were conducted in designed diamond anvil cells with anvil culet of 300 μm, combined with a Solartron-1260 impedance analyzer. A Re gasket was initially pre-indented into a 25 μm thickness of pit, and then its center was drilled by the laser drilling device to form a 200 μm hole. As excellent insulating materials, a mixture powder of epoxy and cubic boron nitride was compressed into the hole, and finally another 100 μm hole was drilled to serve as the insulating sample chamber. For non-hydrostatic EC experiments, we did not use any pressure medium in experiments, while the KCl was adopted for quasi-hydrostatic EC measurements. For the KCl media, it was reported that the pressure gradient is as small as 0.9 GPa at 20 GPa^28^, whose capability to maintain hydrostatic condition is comparable with some liquid media^43^. A ruby was placed on the surface of the electron to determine pressure. AC impedance was acquired in the frequency range of 10^‒1^–10^7 ^Hz. The noble metal platinum foil was selected as the electron material. The collected AC impedance data were analyzed by the Z-View software. Typical impedance spectra were composed of one approximately semicircular arc at high frequencies and another small semicircular arc at low frequencies, which stand for the grain interior and grain boundary contribution. To obtain the grain interior resistance of sample, we only fitted the semicircular arcs at low frequency ranges by the equivalent circuit method. The resistance was then used to calculate the electrical conductivity with the equation:

$$\sigma =L/{RS}$$

where *L* is the thickness of sample (cm), *S* represents electrode cross-sectional area (cm^2^), *R* is fitting resistance (Ω), and σ is sample electrical conductivity (S/cm). The sample thickness *L* under pressure was measured with a micrometer, and the electrode cross-sectional area *S* was ~1.1304×10^-4^ cm^2^.

### In situ synchrotron XRD measurements

High-pressure angular dispersive XRD measurements were carried out at the 13BM-C beamline in Advanced Phonon Source (APS), Argonne National Laboratory (ANL), USA^44^. The symmetrical diamond anvil cell with anvil culet of 300 μm was used to generate high pressure. A 100 μm of hole was drilled in Re gasket as the sample chamber. No pressure medium was used in our XRD measurements in order to generate deviatoric stress. The size of the beam spot was about 15×15 μm, and the X-ray wavelength was 0.4340 Å at the time of experiment. LaB6 was used to calibrate the image plate orientation angles and sample-to-detector distances. The collected XRD diffraction data were integrated with the Dioptas program and later analyzed by the UnitCell software^45^.

### High-pressure Raman scattering measurements

Raman scattering measurements were implemented in symmetrical diamond anvil cells (DACs) with culet size of 250 μm. The sample was loaded into a 100 μm hole in a Re gasket together with a small piece of ruby, which was used as the pressure calibration material. No pressure medium was used in non-hydrostatic Raman experiments, while the mixed solution of methanol and ethanol was chosen to for hydrostatic high-pressure experiment for comparison. Raman spectra were acquired through a micro-confocal Renishaw Raman spectrometer with a 532 nm excitation source, a spectra resolution of -1 cm^-1^ and a grating of 2400 gr/mm. We used a proper laser power of 20 mV to avoid destroying sample structure by the laser energy. The excitation power for ruby fluorescence was 0.5–40 μW. The acquisition time for each spectrum was 120 s to ensure the quantity of spectra. All of Raman spectra were fit by a Gauss function to obtain peak positions.

### Supplementary information

Supplementary Information

## Data Availability

The source datasets for the XRD, Raman and electrical conductivity experiments can be accessed via the 4TU. ResearchData (10.4121/54bf3d3f-e8c2-4e2c-a415-239f71b15506). Any additional data that support the findings of this work are available from the corresponding author upon reasonable request.
