# Supplementary material for: Deviatoric stress-induced metallization, layer reconstruction and collapse of van der Waals bonded zirconium disulfide
Source: Commun Chem. 2024 Jun 22;7:141. doi: 10.1038/s42004-024-01223-1 (PMC11193816; doi:10.1038/s42004-024-01223-1)
Supplement: Supplementary file 1 — Supplementary Information [file 42004_2024_1223_MOESM1_ESM.pdf]

Supplemental Material for "Deviatoric stress-induced metallization, layer reconstruction and collapse of van der Waals bonded zirconium disulfide"

Linfei Yang,[1,2] Junwei Li,[1] Dongzhou Zhang,[3] Yuegao Liu,[4] and Qingyang Hu[1,5]*

[1]*Center for High Pressure Science and Technology Advanced Research, Beijing 100193, China*

[2]*School of Material Science and Engineering, Jingdezhen Ceramic University, Jingdezhen 333001, Jiangxi, China*

[3]*Hawai'i Institute of Geophysics and Planetology, School of Ocean and Earth Science and Technology, University of Hawai'i at Manoa, Honolulu, HI 96822, USA*

[4]*CAS Key Laboratory for Experimental Study under Deep-sea Extreme Conditions, Institute of Deep-sea Science and Engineering, Chinese Academy of Sciences, Sanya 572000, China*

[5]*Shanghai Advanced Research in Physical Sciences (SHARPS), Shanghai 201203, China*

* Corresponding author.

Email addresses: qingyang.hu@hpstar.ac.cn

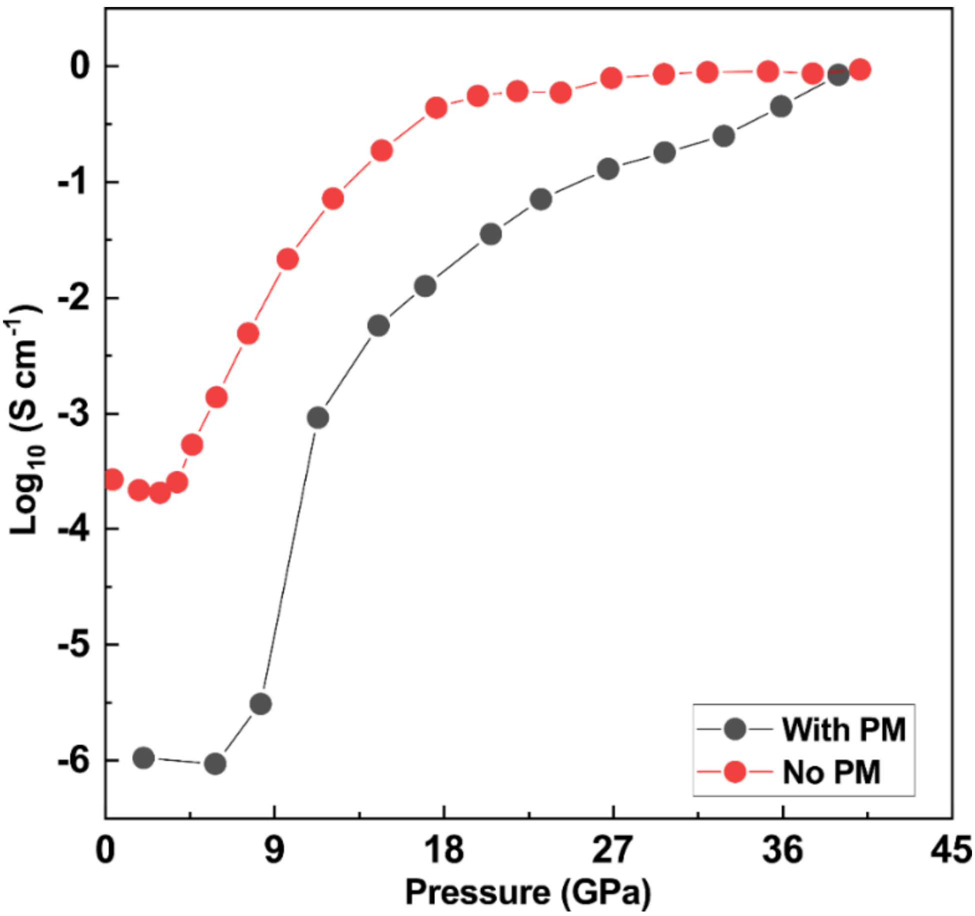

Supplementary Figure 1. Electrical conductivity of ZrS_2 under non-hydrostatic and hydrostatic conditions.

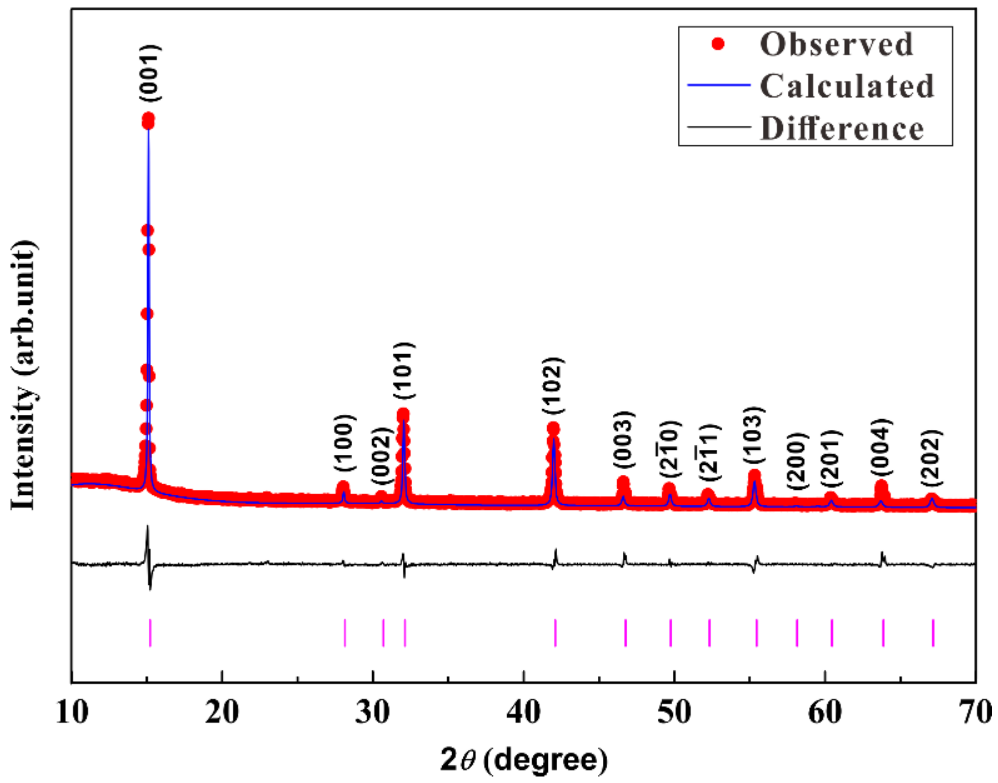

Supplementary Figure 2. The X-ray diffraction data and fitting results for ZrS_2 powder at ambient conditions. The bottom black curve stands for the difference between raw and fitted profile. The raw XRD pattern is fitted by Rietveld profiles. The wavelength of x-ray is 1.5406 Å. The *R*-factor is wR_1 = 7.822 %. The magenta vertical marks denote the calculated positions for the Bragg reflections.

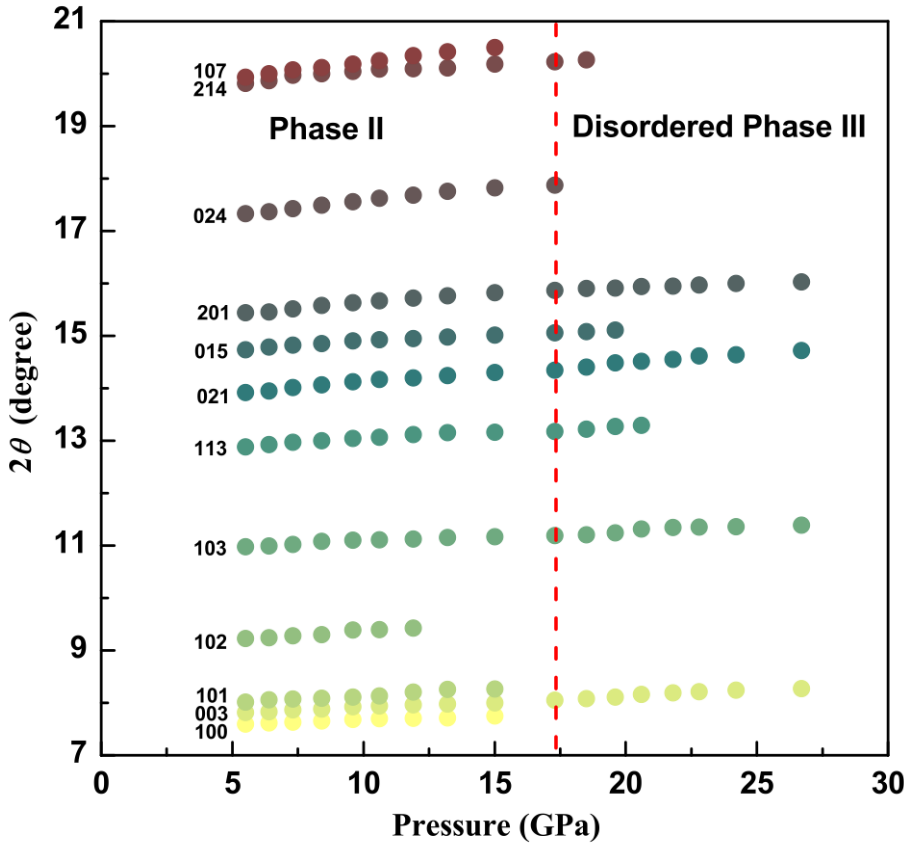

Supplementary Figure 3. The pressure dependence of the collected 2θ angles of diffraction peaks. Most diffraction peaks disappeared in the disordered phase III.

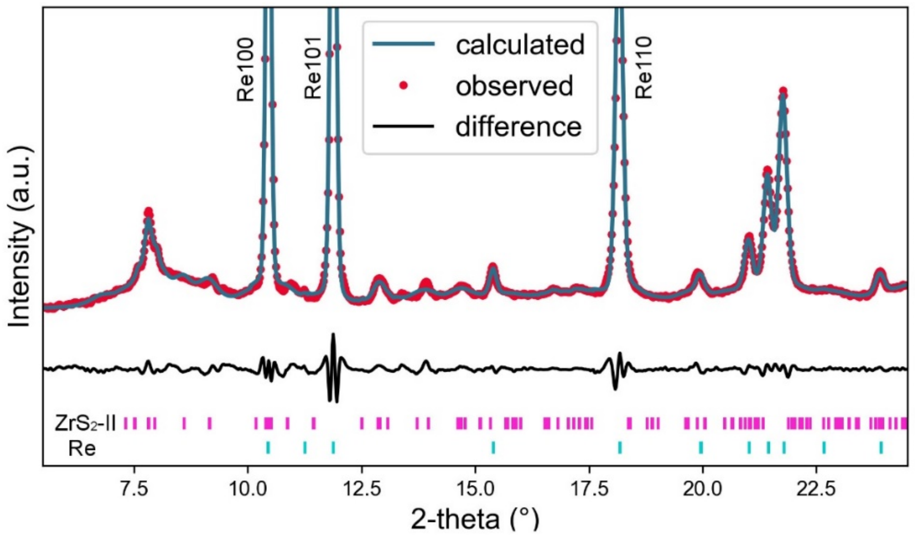

Supplementary Figure 4. *LeBail* refinement of ZrS_2-II. The space group is *Pmm*2 with lattice parameters: a = 3.297 (6), b = 3.627 (7), c = 9.509 (11). The refinement factors after removing background are R_1 = 0.048, wR_1 = 0.077. The wavelength of the incident x-ray is 0.4344 Å.

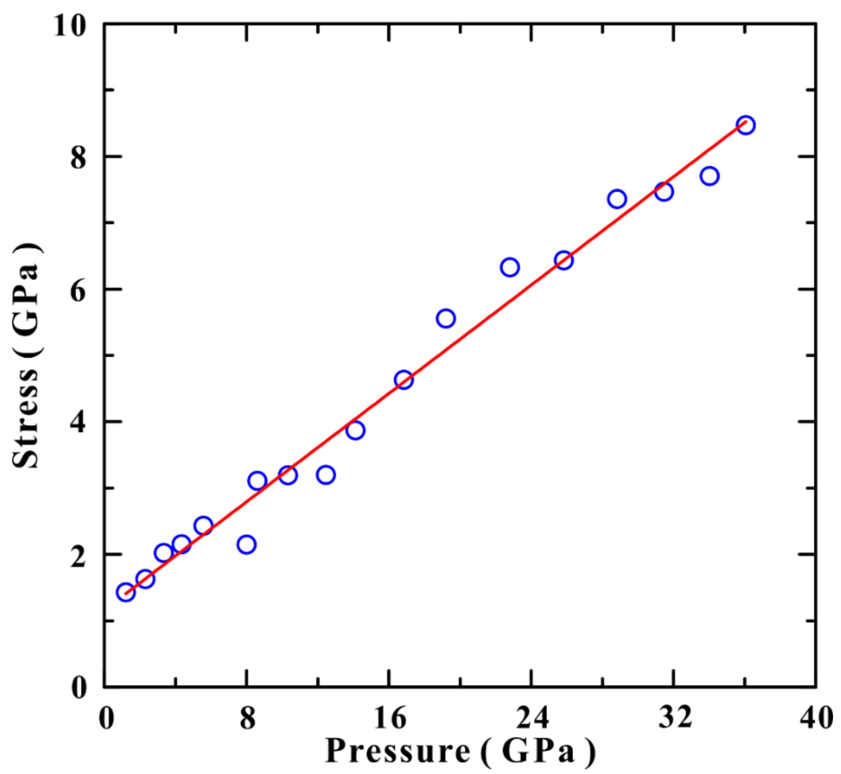

Supplementary Figure 5. The uniaxial stress of sample at the pressure range of 0–40 GPa. The error bars are located within the symbols.

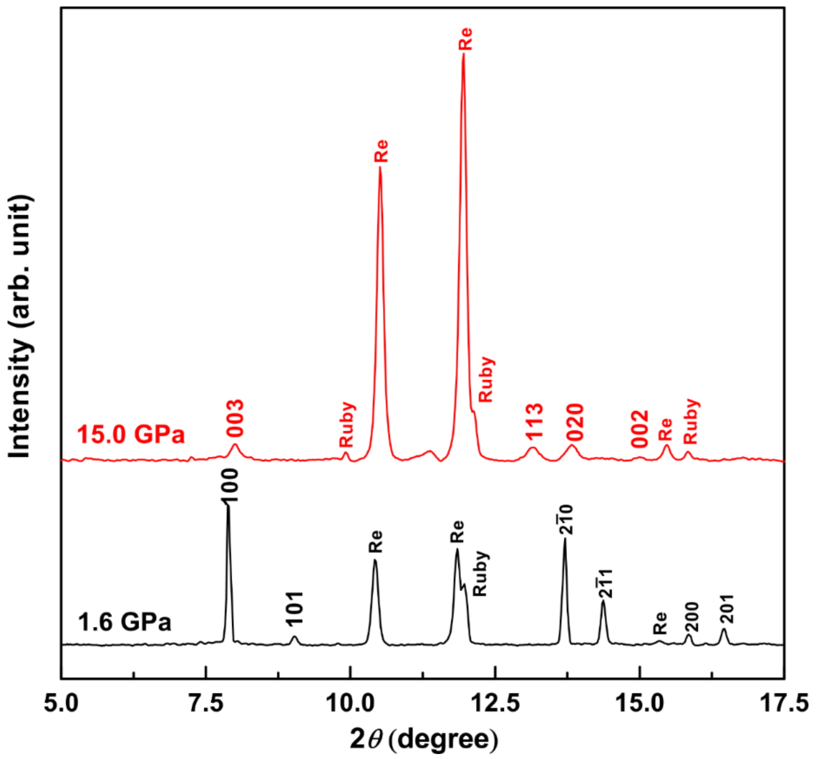

Supplementary Figure 6. Full spectra at collected at 1.6 GPa and 15.0 GPa. The Miller indices (hkl) are drawn in diagram for $P\bar{3}m1$ and $Pmm2$ phases.

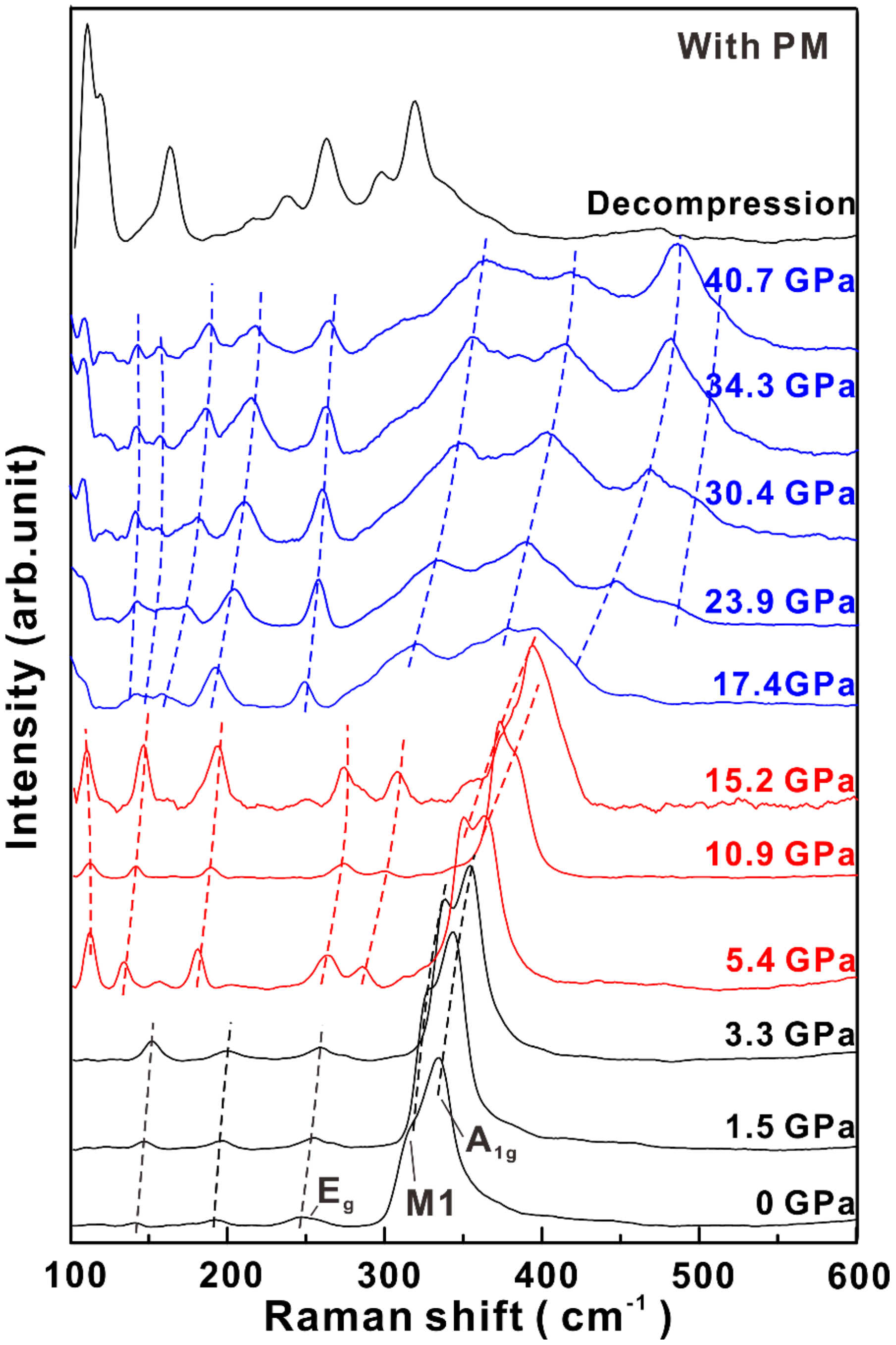

Supplementary Figure 7. Raman results of 1T-ZrS_2 as a function of pressure under quasi-hydrostatic conditions. The uppermost data corresponds to the decompressed Raman spectrum at 1 atm. The dashed lines represent the shift tendency of phonon modes.

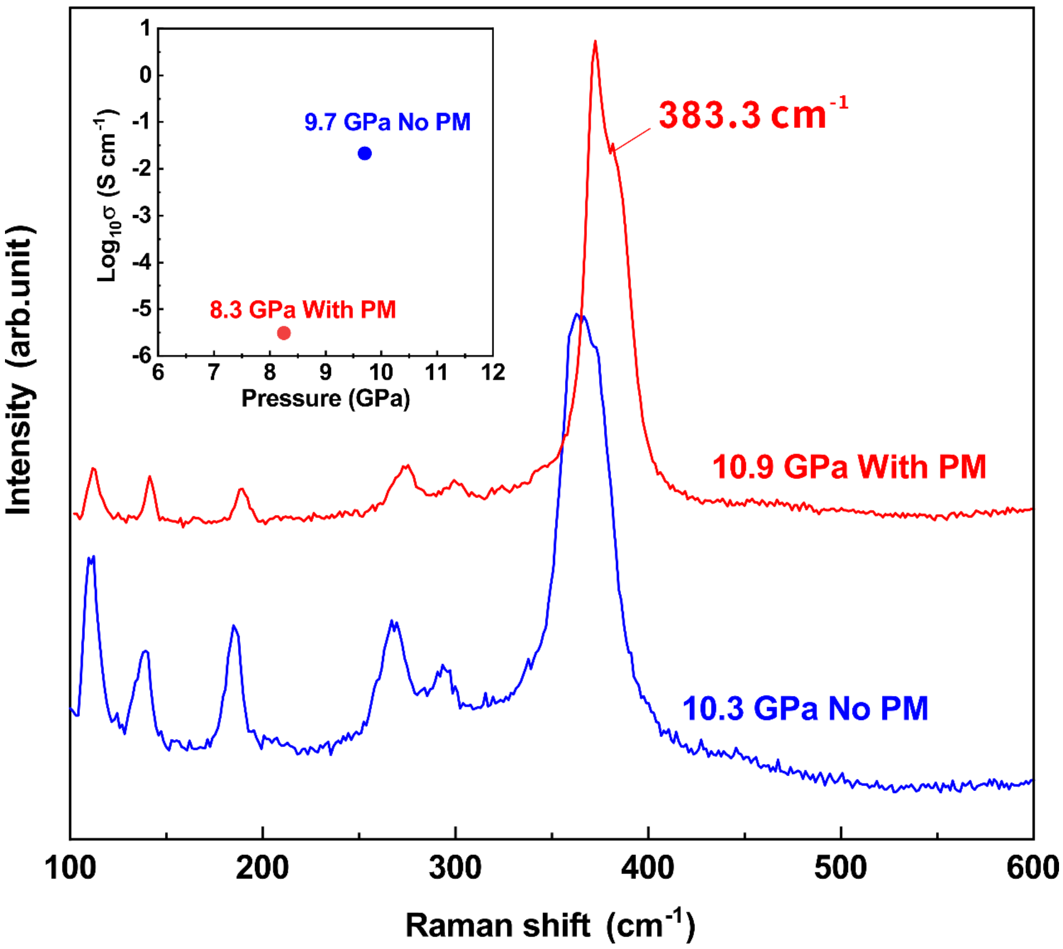

Supplementary Figure 8. Raman spectra of phase II under non-hydrostatic pressure of 10.3 GPa (no PM) and hydrostatic pressure of 10.9 GPa (with PM). Inset: electrical conductivity of phase II at non-hydrostatic pressure of 9.7 GPa and hydrostatic pressure of 8.3 GPa.
